# The effect of dietary nitrate on weight management: a systematic review and meta-analysis

**DOI:** 10.3389/fpubh.2026.1798811

**Published:** 2026-04-28

**Authors:** Wenjun Cai, Peng Wei, Fan Yang, Lin Shu, Yongyao Du, Huirong Feng, Jiaojiao Shi, Chao Kang, Xiaoli Peng

**Affiliations:** 1 Office of Quality Management, The General Hospital of Western Theater Command, Chengdu, Sichuan, China; 2 School of Medicine, Macau University of Science and Technology, Macau, China; 3 School of Public Health, Chengdu Medical College, Chengdu, Sichuan, China; 4 Department of Medical Data, The General Hospital of Western Theater Command, Chengdu, Sichuan, China; 5 Department of Clinical Nutrition, The General Hospital of Western Theater Command, Chengdu, Sichuan, China; 6 Sichuan Provincial Key Laboratory of Philosophy and Social Sciences for Intelligent Medical Care and Elderly Health Management, Chengdu Medical College, Chengdu, Sichuan, China

**Keywords:** dietary nitrate, meta-analysis, metabolic marker, obesity, weight management

## Abstract

**Background:**

Obesity has emerged as a significant global public health concern. Despite its potential therapeutic benefits, the existing evidence regarding dietary nitrate supplementation remains inconclusive. This systematic review and meta-analysis aimed to evaluate the effects of dietary nitrate intake on obesity-related outcomes.

**Methods:**

A comprehensive search of randomized controlled trials (RCTs) investigating nitrate supplementation in adults with obesity was conducted across PubMed, Scopus, Web of Science, and Embase up to April 2026. The primary outcomes included body weight, body mass index (BMI), blood pressure, and lipid-related markers. The study adhered to the PRISMA 2020 guidelines for reporting.

**Results:**

Eight RCTs involving 199 participants were included in the meta-analysis. No significant effects of nitrate supplementation were observed on body weight, BMI, or diastolic blood pressure (DBP). A statistically significant reduction was observed in systolic blood pressure (SBP, 95% CI: −0.63 to −0.01; SMD = −0.32, *p* = 0.04; *I^2^* = 41%). In addition, dietary nitrate supplementation was associated with significant reductions in total cholesterol (TC, 95% CI: −1.11 to −0.06; SMD = −0.59, *p* = 0.03; *I^2^* = 50%) and triglycerides (TG, 95% CI: −0.81 to −0.09; SMD = −0.45, *p* = 0.01; *I^2^* = 0%). Subgroup analysis stratified by intervention duration revealed no significant interaction effects for either SBP or DBP (all *P* for interaction > 0.05). Publication bias was detected for BMI and DBP.

**Conclusion:**

Dietary nitrate supplementation exerts favorable effects on lipid parameters and systolic blood pressure, with no remarkable impacts on body weight and BMI observed. Given the relatively small sample size of the included evidence, future large-sample, high-quality, long-term randomized controlled trials are essential to confirm and reinforce the present results.

**Systematic review registration:**

https://www.crd.york.ac.uk/PROSPERO/, CRD420251078542.

## 1 Introduction

Obesity has emerged as a critical global public health concern, with its prevalence consistently rising in both developed and developing countries. The World Health Organization (WHO) identifies overweight and obesity as significant contributors to numerous chronic non-communicable diseases, including type 2 diabetes, cardiovascular disorders and certain malignancies (1). The increasing health and economic burdens associated with obesity underscore the urgent need for safe and effective weight management strategies (2). Dietary nitrate, primarily derived from vegetables such as spinach, lettuce, and beetroot, serves as a major source of nitrate intake in humans (3, 4). Due to its high nitrate concentration, beetroot juice has been widely utilized as a supplementation medium in clinical investigations (5). Nitrate supplementation has been linked to various health benefits, including enhanced cardiovascular function, improved exercise performance, and anti-inflammatory properties (6, 7). Nitric oxide, a well-characterized signaling molecule, exerts vasodilatory effects and plays a regulatory role in energy metabolism (8, 9). Emerging research indicates that nitrate-derived nitric oxide may promote lipid oxidation and energy expenditure, suggesting a potential mechanism for weight regulation (10, 11). This proposed mechanism has generated increasing interest in dietary nitrate as a potential intervention for obesity management (12).

However, results from human studies remain inconsistent. While some clinical trials report beneficial effects of dietary nitrate or nitrate-rich foods (e.g., beetroot juice, leafy vegetables) on metabolic parameters and body composition (13, 14), others have not found significant alterations (15, 16). These discrepancies may stem from variations in study populations, intervention durations, and administered nitrate doses. Given these uncertainties, we conducted a systematic review and meta-analysis to consolidate existing evidence on the impact of dietary nitrate on weight regulation. By synthesizing data from preprint repositories and academic databases, this study aims to (i) critically evaluate available clinical trials, (ii) elucidate potential sources of heterogeneity, and (iii) provide evidence-based recommendations regarding the feasibility of dietary nitrate as a nutritional strategy for obesity prevention and treatment.

## 2 Methods

### 2.1 Search strategy

This systematic review was conducted in accordance with the guidelines outlined in the Preferred Reporting Items for Systematic Reviews and Meta-Analyses (PRISMA) statement and the Cochrane Handbook for Systematic Reviews (17). A systematic search was conducted in MEDLINE (via PubMed), Scopus (via Elsevier), Web of Science [limited to the Science Citation Index Expanded (SCIE) and Social Sciences Citation Index (SSCI), via Clarivate], and Embase (via Elsevier) in April 2026, without any restrictions on language or publication year. The restriction to SCIE and SSCI was applied to ensure the inclusion of high-quality, peer-reviewed studies and to enhance the methodological rigor of the evidence base. The search included the following keywords: “beetroot,” “beetroot juice,” “nitrates,” “nitrites,” “dietary nitrate,” “obesity,” “overweight,” “body weight” and “weight management” to ensure comprehensive coverage of nitrate-related interventions. Beetroot was included as a representative and commonly studied source of dietary nitrate rather than the sole focus of the search strategy. No language restrictions were applied during the literature search. Validated filters were applied to identify RCTs. The detailed search strategies for all databases are provided in Supplementary Table 1. Additionally, a manual search was conducted on preprint platforms (medRxiv and Research Square) and other databases (CINAHL, China National Knowledge Infrastructure databases, Wanfang Database, and Scielo). Non-English literature was translated using either translation tools or professional assistance. To supplement our searches of academic databases, additional searches were conducted in gray literature sources, including ProQuest Dissertations and Theses, Ethos, and ClinicalTrials.gov. The meta-analysis has been registered on PROSPERO (CRD420251078542).

### 2.2 Eligibility criteria

Inclusion criteria were defined according to the PICOS framework: (1) Participants: Adults (age ≥18 years) classified as overweight or obese; (2) Study design: randomized controlled trials (RCTs); (3) Intervention: dietary nitrate supplementation or nitrate-rich foods; (4) Comparator: placebo or control group; (5) Outcomes: studies reporting quantitative data for weight-related outcomes, including body weight, BMI, fat mass (FM) and waist circumference (WC), SBP, DBP, TC, or TG; (6) Data availability: studies providing sufficient methodological details and outcome data for meta-analysis. Studies were excluded if they met any of the following criteria: (1) duplicate publications; (2) animal or cell-based studies; (3) studies with irretrievable outcome data despite attempts to contact the authors; (4) secondary studies (e.g., reviews or meta-analyses); (5) studies lacking sufficient methodological detail or complete outcome reporting (e.g., conference abstracts).

### 2.3 Screening and data extraction

A standardized data collection form was developed in Microsoft Excel. Two independent reviewers (FY and PW) screened studies retrieved from electronic databases to identify relevant studies. Irrelevant titles were excluded, and the remaining articles were systematically screened for eligibility based on their abstracts and full texts. Data were extracted from the included studies, including information presented in their figures and tables. In addition, when BMI values were not reported, they were calculated from body weight and height using the formula BMI = weight (kg)/height^2^ (m^2^), and the corresponding standard deviations were derived accordingly. If data were missing, the corresponding author was contacted via email to request the missing information; if the required data could not be obtained, the study was excluded. The extracted data included the title, authors, year, country, sample size, age, type and dose of nitrate, duration of intervention, outcomes, body weight, BMI, SBP, DBP, TC, TG, FM and WC were predefined as exploratory secondary outcomes in the present analysis. Relevant data were systematically extracted during literature screening according to the prespecified protocol.

### 2.4 Quality assessment

The risk of bias of the included studies was independently assessed by two investigators (WC, FY), with cross-validation of the results. The revised Cochrane Risk of Bias tool (RoB 2.0) was used to evaluate the methodological quality of the included randomized controlled trials (18). This tool assesses five domains: bias arising from the randomization process, bias due to deviations from intended interventions, bias due to missing outcome data, bias in measurement of the outcome, and bias in selection of the reported result. Each domain was judged as “low risk of bias”, “some concerns”, or “high risk of bias”, and an overall risk-of-bias judgment was assigned for each study according to the RoB 2.0 algorithm (19). The remaining studies exhibited minor concerns in specific areas but maintained a high level of methodological integrity overall. Per RoB 2.0 guidelines, overall risk-of-bias judgments for each study were initially generated using the algorithm-based approach. When these judgments were inconsistent with methodological quality or relevance to primary outcomes, reviewers reclassified the ratings accordingly: two studies were reclassified from some concerns to low risk, and one study was reclassified from high risk to some concerns. All adjustments were documented and justified in the results, with final judgments presented in the risk-of-bias figures. Disagreements between reviewers were resolved through discussion.

### 2.5 Statistical analysis

A meta-analysis of the included studies was conducted using Review Manager v.5.4 (The Cochrane Collaboration, Copenhagen, Denmark) and STATA v.17.0 software (College Station, TX: StataCorp LLC). Effect sizes for continuous variables were expressed as SMD and 95% CIs, which were analyzed as summary statistics. To ensure comparability across studies, data presented as medians and interquartile ranges (IQR) were converted to means and standard deviations (SD) using the Hozo method (20). For variables with reported standard errors (SE), the SD was calculated using the following formula: SE × √(sample size). Post-intervention outcome measures (body weight, BMI, SBP, DBP, TC, TG) were selected for further analysis. Subgroup analysis was conducted based on intervention duration (≥2 weeks vs. <2 weeks) to explore potential sources of heterogeneity. Specifically, the analysis focused on systolic blood pressure (SBP) and diastolic blood pressure (DBP), outcomes for which intervention duration may play a significant role. Other subgroup analyses (e.g., age and BMI) were not performed due to the limited number of studies available for each outcome. Sensitivity analyses and formal assessments of publication bias (Egger’s and Begg’s tests) were conducted only when sufficient studies were available. For outcomes with fewer studies (e.g., TC and TG), these analyses were not performed to avoid unreliable estimates. For crossover trials, outcome data from the intervention and control phases were extracted and included in the meta-analysis according to recommended approaches for crossover designs. The DerSimonian and Laird method was employed to estimate the between-study variance. Random-effects models were used based on the observed heterogeneity of study outcomes. Heterogeneity among studies was assessed using the I^2^ statistic. I^2^ values of <25%, 25–50, and >50% were considered low, moderate, and high heterogeneity, respectively. If *I^2^* ≥ 50% and *p*-value < 0.1, the outcome was considered to exhibit significant heterogeneity. Publication bias was evaluated through funnel plots, Begg’s rank correlation, and Egger’s weighted regression test. If funnel plot asymmetry was detected, the “trim-and-fill” method was employed to estimate potentially missing studies.

## 3 Results

### 3.1 Search results

**Figure 1:**
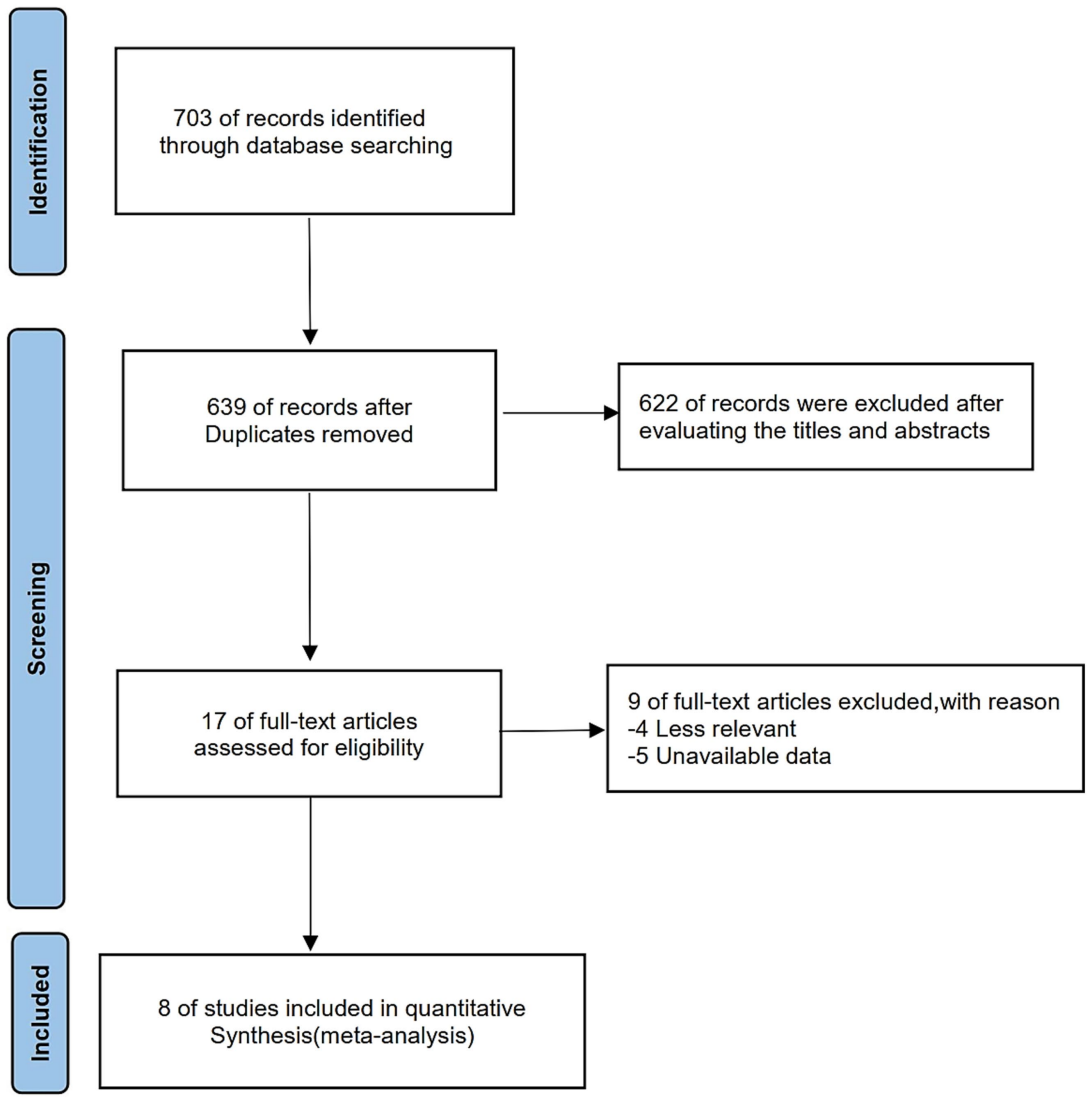
Flow diagram for identification of studies in the systematic review.

A total of 703 articles were identified through comprehensive database searches, with 64 duplicates excluded. After screening based on titles and abstracts, 622 articles were excluded, resulting in 17 articles eligible for full-text evaluation. Among these, 4 studies were excluded because they were irrelevant to the objectives of this meta-analysis, and 5 were excluded because they lacked essential outcome data. Consequently, 8 RCTs were ultimately included in the meta-analysis, all of which were peer-reviewed publications (Figure 1 and Supplementary Table 2).

### 3.2 Study characteristics

The eight included studies were published between 2014 and 2023 and conducted in five countries (the United Kingdom, Iran, Australia, Italy, and the Netherlands). All studies randomly allocated participants to placebo, control, or intervention groups to minimize allocation bias. Participants across these studies were exclusively obese adults aged 18 to 80 years. The intervention consisted of nitrate supplementation (primarily through beetroot juice), whereas control groups received corresponding placebos. The study populations consisted predominantly of obese adults, with a predominance of male participants and individuals in middle to older age groups. Intervention durations ranged from 7 days to 13 weeks (Table 1).

**Table 1:** Characteristics of the studies included in this review.

First author, year	Country	Participant numbers (n)	Age range (years)	Duration	Population	Intervention	Findings
Ashor et al. (2014) (21)	UK	21	55–70 years	4 weeks	In the experimental group, there were 7 men and 3 women; in the control group, 5 men and 6 women were enrolled. Non-smoking overweight or obese individuals aged 55–70 years with a BMI of 25–40 kg/m^2^.	Daily supplementation with beetroot juice concentrate (~300–400 mg nitrate per bottle), compared with low-nitrate blackcurrant juice (<5 mg nitrate).	Beetroot supplementation significantly elevated salivary and urinary nitrate levels compared with blackcurrant, but no significant differences were observed.
Asgary et al. (2016) (22)	Iran	24	25–68 years	2 weeks (intermitted by a 2-week washout period)	The study enrolled adult subjects aged 25–68 years with SBP of 130–139 mmHg or DBP of 85–89 mmHg.	Daily consumption of either 250 mL raw beetroot juice or 250 g cooked beetroot for 2 weeks (non-placebo-controlled design).	Short-term supplementation with RBJ or CB improved blood pressure, endothelial function, and systemic inflammation, with RBJ showing superior effects on the latter two outcomes.
Wong et al. (2014) (28)	Australia	37	18-80 years	12 weeks	The study sample comprised 20 men and 17 women. Eligible participants were adults with untreated high-normal or borderline hypertension and BMI 20–35 kg/m^2^.	Daily supplementation with a combined nutraceutical containing 500 mg olive leaf extract, 100 mg green coffee bean extract, and 150 mg beetroot powder.	6 weeks of daily supplementation showed no effect on 24-h ambulatory or clinic blood pressure, blood lipids, glucose, or insulin sensitivity in adults with borderline or mildly elevated blood pressure.
Alharbi et al. (2023) (23)	Italy	29	50–75 years	14 days	In the experimental group, there were 7 men and 22 women; in the control group, 3 men and 11 women were enrolled. The mean age was 61.3 ± 5.9 years, ranging from 52 to 74 years. The mean BMI was 34.5 ± 5.8 kg/m2.	Daily intake of 70 mL concentrated beetroot juice for 14 days in combination with calorie restriction.	Dietary nitrate combined with calorie restriction may enhance vascular and cognitive function in overweight older adults, without worsening weight loss–related declines in energy metabolism.
Smeets et al. (2020) (25)	The Netherlands	18	40–70 years	The study consisted of five test days, each separated by a wash-out period of at least 1 week.	All participants in this study were male. Eligible participants were men aged 40–70 years with WC ≥ 102 cm, stable weight, no antihypertensive or metabolic drugs.	Single-dose interventions including: (i) 15 g beetroot powder; (ii) 15 g beetroot powder + 0.8 g L-arginine; (iii) 15 g beetroot powder + 1.5 g L-arginine; or (iv) 3 g L-arginine alone, administered across separate test days with washout periods.	A single dose of beetroot powder combined with L-arginine did not improve postprandial endothelial function in abdominally obese men.
Lara et al. (2015) (16)	UK	30	55–70 years	7 days	*n* = 20 with available data for meta-analysis. The study included healthy, non-smoking men and women aged 55–70 years with a BMI of 25–40 kg/m^2^.	Consumption of 70 mL concentrated beetroot juice twice daily (~600 mg nitrate/day) for 7 days, compared with intermittent high-intensity exercise.	Consumption of IHGE or beetroot juice for 7 days did not significantly affect blood pressure or peripheral arterial function in overweight and obese middle-aged and older adults.
Smeets et al. (2022) (26)	The Netherlands	18	18–60 years	5 months, 4h intervention	All participants in this study were male. The 4-h intervention included adults aged 18–60 years with WC ≥ 102 cm, stable weight, no relevant medications or metabolic/cardiovascular disease.	Acute administration of a nitrate-rich drink containing 10 mmol (~625 mg) potassium nitrate dissolved in 30 mL water.	This 5-month trial did not provide evidence that a single dose of inorganic nitrate affects 4-h vascular endothelial function in abdominally obese men.
Babateen et al. (2023) (15)	UK	MN:17 PL:15	60–75 years	13 weeks	The effective experimental group consisted of 17 participants. The intervention lasted 13 weeks and included overweight or obese older adults aged 60–75 years with a body mass index of 25–40 kg/m^2^.	Daily supplementation with concentrated beetroot juice at varying nitrate doses: high (~400 mg/day; 70 mL twice daily), medium, or low dose (70 mL once daily).	Thirteen-week supplementation with medium and low, but not high, nitrate doses improved blood pressure and endothelial function in older overweight and obese adults.

RCT, Randomized controlled trials.

### 3.3 Subject characteristics

A total of 199 unique participants were included in the meta-analysis. Due to the crossover design adopted in some studies, data were contributed by participants during both the intervention and control phases, resulting in 296 observations when counted across groups. Of these, 50.34% were assigned to the intervention (nitrate) group, whereas 49.66% were assigned to the control group. The majority of participants were male, and all had been diagnosed with obesity. The study population consisted of adults over 18 years of age, with a notable proportion of older individuals (Table 1).

### 3.4 Risk of bias

**Figure 2:**
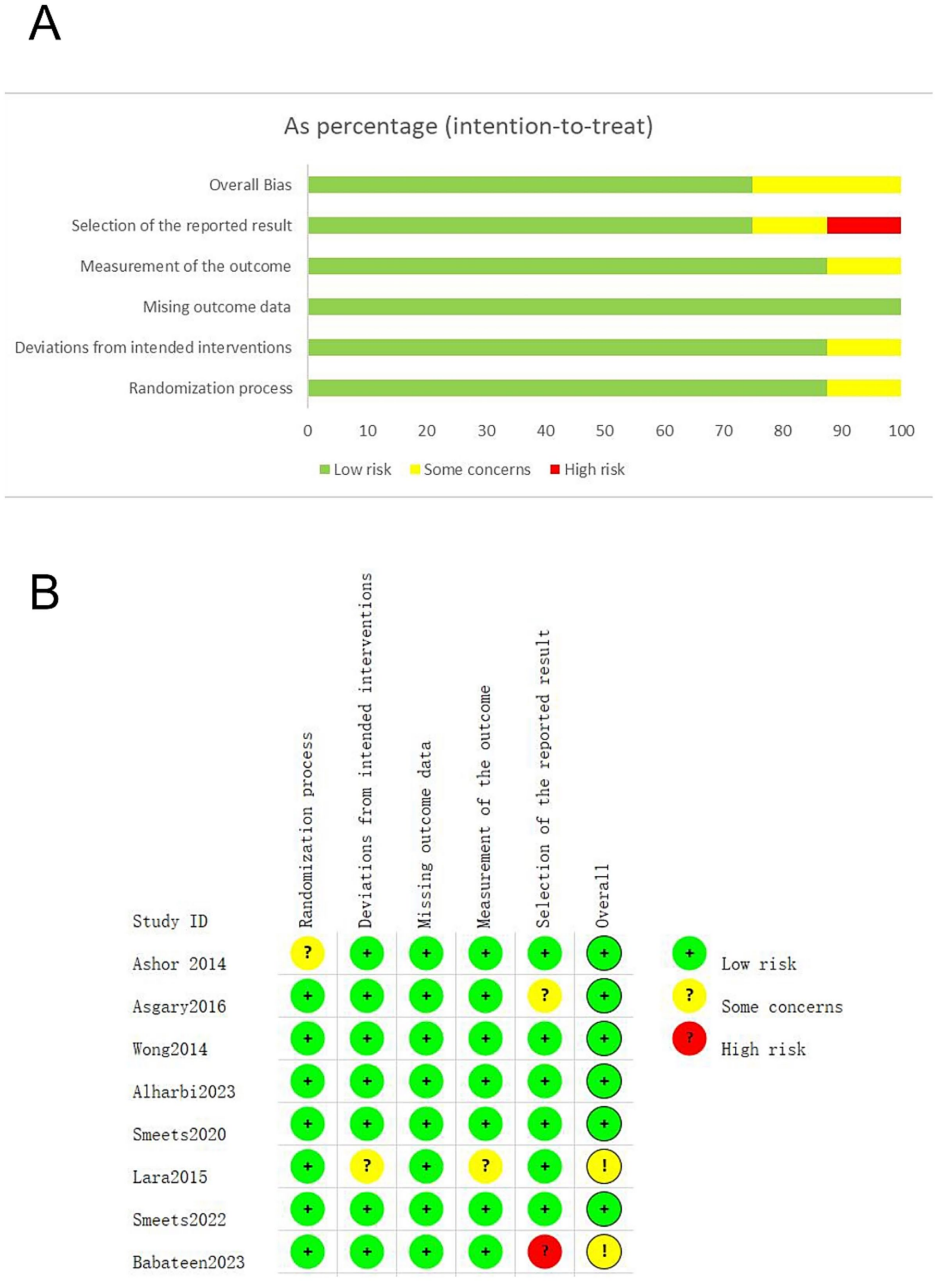
Risk of bias (RoB) analysis highlighting results in all domains examined within the eight identified studies **(A)** and overall risk of bias for included studies **(B)**.

The risk of bias of the included studies was assessed using the revised Cochrane Risk of Bias tool (RoB 2.0), covering five domains: the randomization process, deviations from intended interventions, missing outcome data, measurement of the outcome, and selection of the reported result. According to the RoB 2.0 guidance, overall risk-of-bias judgments were derived using the algorithm-based approach, with additional reviewer assessment applied when necessary to ensure consistency with methodological quality and relevance to the primary outcomes. Overall, six studies were judged to be at low risk of bias, and two studies were rated as having some concerns, with no studies classified as high risk of bias. In three studies, the algorithm-based overall judgments were further reviewed and reclassified. Specifically, Ashor et al. (21) and Asgary et al. (22) were reclassified from some concerns to low risk, as identified issues were limited to outcomes not central to the review objective and did not affect the primary weight-related results. Babateen et al. (15) was reclassified from high risk to some concerns, since the high-risk judgment was confined to non-primary outcome reporting, while all domains related to the primary outcomes were assessed as low risk. The majority of studies demonstrated adequate methodological quality, with low risk of bias across most domains. At the domain level, all included studies were judged to be at low risk of bias for missing outcome data. Similarly, the domains of measurement of the outcome and deviations from intended interventions were predominantly rated as low risk, with only a small proportion of studies showing some concerns. For the randomization process, most studies were assessed as low risk, although one study raised some concerns due to insufficient reporting. Notably, the domain of selection of the reported result presented the greatest potential source of bias, with one study judged as high risk and another study as having some concerns. These issues were primarily related to lack of pre-specified analysis plans or insufficient reporting transparency. A detailed summary of the risk-of-bias assessment is presented in Figure 2 and Supplementary Table 3. These methodological limitations should be considered when interpreting the pooled results.

### 3.5 Parameters of weight management

**Figure 3:**
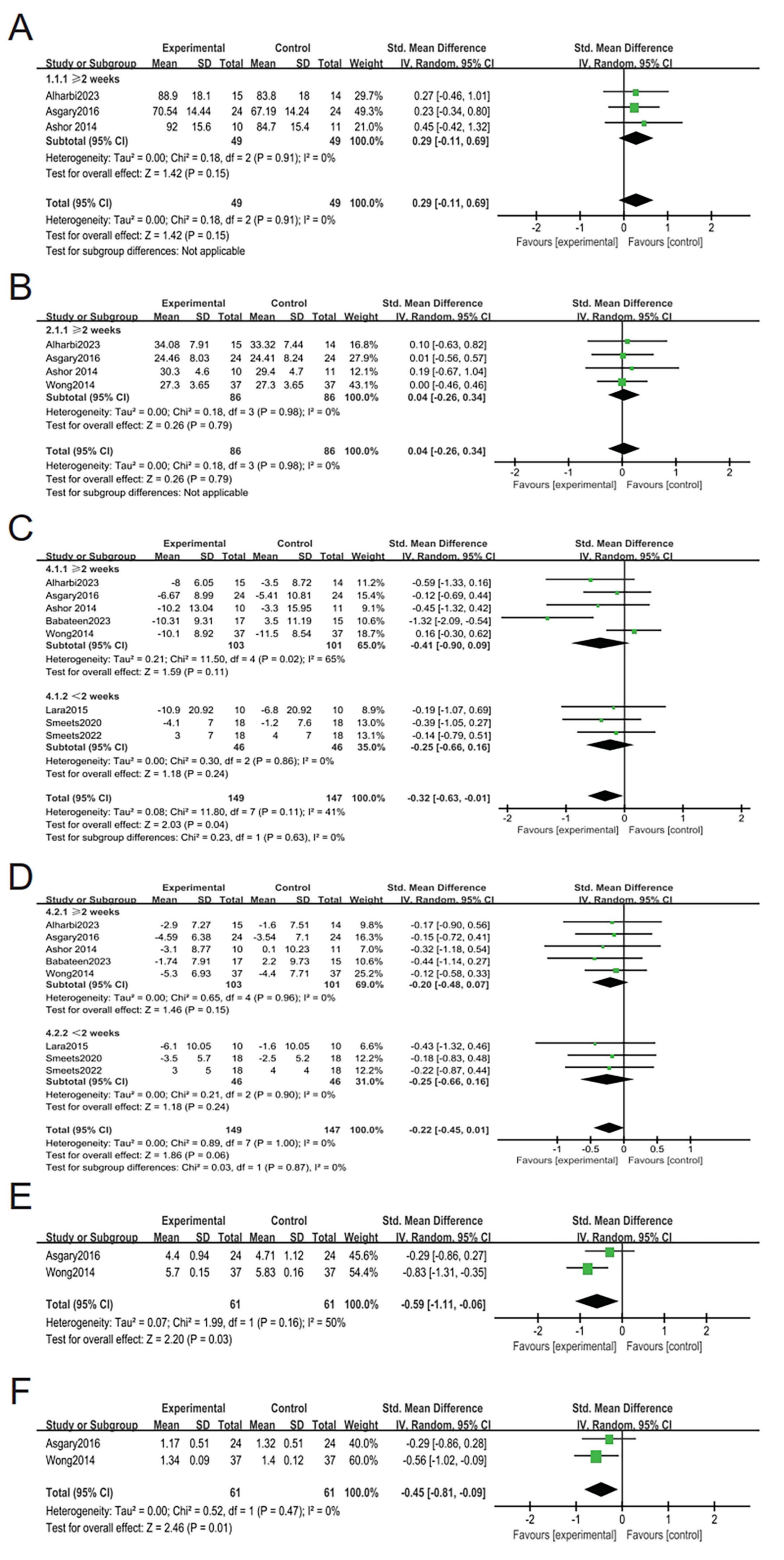
Forest plots displaying the standardized mean differences (SMDs) and 95% confidence intervals for the effects of the intervention on body weight **(A)**, BMI **(B)**, SBP **(C)**, DBP **(D)**, serum TC **(E)**, and TG **(F)**.

The meta-analysis demonstrated that nitrate supplementation had no significant effects on body weight (95% CI: −0.11 to 0.69; SMD = 0.29, *p* = 0.15; I^2^ = 0%, Figure 3A), BMI (95% CI: −0.26 to 0.34; SMD = 0.04, *p* = 0.79; I^2^ = 0%, Figure 3B), or DBP (95% CI: −0.45 to 0.01; SMD = −0.22, *p* = 0.06; *I^2^* = 0%, Figure 3D). However, a statistically significant reduction was observed in SBP (95% CI: −0.63 to −0.01; SMD = −0.32, *p* = 0.04; *I^2^* = 41%, Figure 3C). For lipid-related outcomes, dietary nitrate supplementation was associated with significant reductions in TC (95% CI: −1.11 to −0.06; SMD = −0.59, *p* = 0.03; I^2^ = 50%, Figure 3E) and TG (95% CI: −0.81 to −0.09; SMD = −0.45, *p* = 0.01; I^2^ = 0%, Figure 3F). However, these results were derived from a small number of studies and should be interpreted with caution. Extractable data on FM and WC were reported by only one included trial (23). Due to the insufficient number of datasets, a quantitative pooled meta-analysis for the above two adiposity-related outcomes was not feasible in the present analysis.

The overall pooled analysis demonstrated a statistically significant reduction in SBP (*p* = 0.04, Figure 3), whereas no significant effects were observed for body weight, BMI, or DBP (all *p* > 0.05). In addition, significant reductions were identified for lipid-related parameters, including total cholesterol (TC) (*p* = 0.03) and triglycerides (TG) (*p* = 0.01). Subgroup analyses stratified by intervention duration (≥2 vs. <2 weeks) did not reveal any statistically significant differences between subgroups (all P for interaction > 0.05), suggesting that the duration of nitrate supplementation may not substantially influence the observed outcomes within the available evidence. Although statistically significant effects were observed for SBP, TC, and TG in the overall analysis, these findings were derived from a limited number of studies. Therefore, they should be interpreted with caution. Taken together, these results suggest that dietary nitrate supplementation may exert modest benefits on cardiovascular and lipid-related parameters, while having limited impact on body composition. However, given the small sample size and limited number of included studies, the current evidence remains insufficient to draw definitive conclusions.

Collectively, these findings suggest that dietary nitrate supplementation may confer modest benefits on systolic blood pressure as well as lipid-related parameters, including TC and TG. However, no consistent effects were observed for anthropometric outcomes such as body weight and BMI. Subgroup analyses based on intervention duration did not reveal significant differences, indicating that the observed effects were relatively consistent across study durations. Given that these findings are derived from a limited number of randomized controlled trials with relatively small sample sizes, the current evidence remains insufficient to support definitive conclusions.

### 3.6 Publication bias

**Figure 4:**
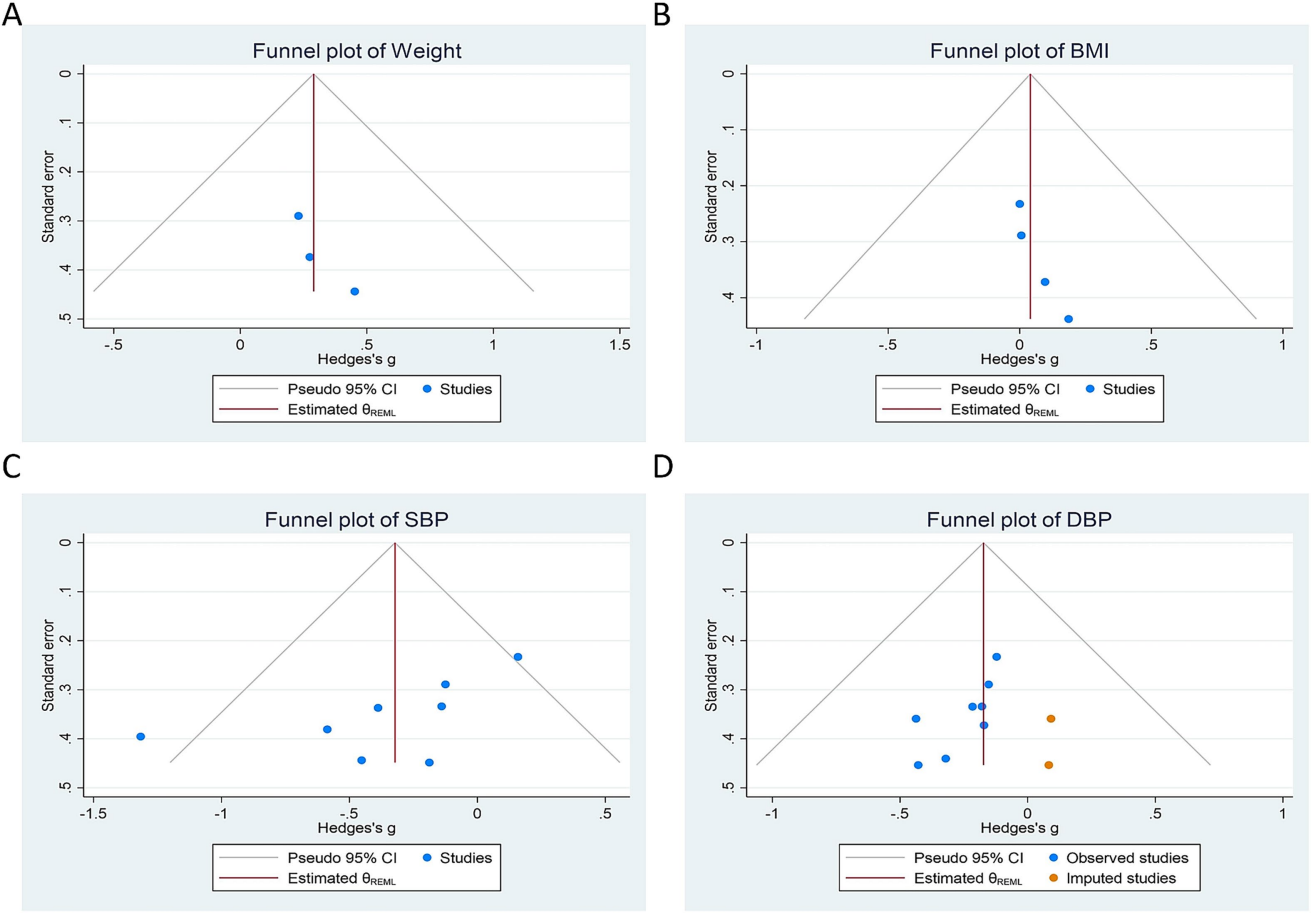
Funnel plot detailing publication bias in the studies reporting the impact on body weight **(A)**, BMI **(B)**, SBP **(C)**, and DBP **(D)** levels.

Funnel plot analysis revealed slight asymmetry in the assessments of body weight, BMI, SBP, and DBP. Following trim-and-fill adjustment, the analyses suggested potential missing studies (0, 0, 0 and 2 studies, respectively), indicating a limited impact of publication bias in this meta-analysis. These findings are illustrated in Figure 4. The results of Egger’s regression tests and Begg’s rank correlation analyses indicated evidence of publication bias for BMI and DBP and are detailed in Table 2. Sensitivity analyses using a leave-one-out approach demonstrated that the pooled estimates remained stable.

**Table 2:** Imputed effect sizes and the results of Begg’s rank correlation and Egger’s regression tests in the meta-analysis of weight management-related outcomes.

Outcome	*n* ^a^	SMD	95%CI	*p*-value^b^	*p*-value^c^
Weight	0	0.290	−0.109 to 0.689	0.2963	0.252
BMI	0	0.040	−0.259 to 0.339	0.0894	0.049
SBP	0	−0.322	−0.634 to −0.010	0.0635	0.059
DBP	2	−0.173	−0.384 to 0.038	0.0354	0.026

a The number of imputed studies according to the trim and fill correction method.

b Begg’s rank correlation test.

c Egger’s weighted regression test.

## 4 Discussion

This meta-analysis systematically evaluated the effects of dietary nitrate supplementation on weight management by synthesizing data from eight RCTs involving 199 participants. The findings revealed no statistically significant effects of nitrate supplementation on key anthropometric parameters, including body weight and BMI. Notably, a statistically significant reduction in SBP was observed in the pooled analysis. In addition, significant reductions were identified in lipid-related parameters, including total cholesterol (TC) and triglycerides (TG), suggesting that the primary physiological benefits of dietary nitrate may be mediated through cardiovascular and metabolic pathways rather than direct effects on body composition (24).

Subgroup analyses based on intervention duration did not demonstrate statistically significant differences for SBP and DBP, suggesting that the effects were not modified by study duration. Unlike previous analyses stratified by age or BMI, which are often limited by small sample sizes, the current findings do not support a clear modifying effect of these factors. Although prior randomized controlled trials have consistently reported the blood pressure–lowering effects of nitrate supplementation [e.g., Babateen et al. (15) and Asgary et al. (22)], evidence regarding its influence on lipid metabolism remains limited. Therefore, the observed reductions in TC and TG in this analysis may provide preliminary insights into the broader cardiometabolic effects of dietary nitrate, although these findings should be interpreted cautiously.

The current analysis revealed no significant alterations in anthropometric parameters, including body weight, BMI, or DBP, despite the potential metabolic effects of nitrate-derived nitric oxide (NO). This lack of observable effects may be attributed to several methodological and physiological factors. First, the majority of included trials employed relatively short intervention durations (7 days to 13 weeks) (21), which may be inadequate to induce detectable changes in body composition or long-term energy homeostasis. Second, substantial heterogeneity existed in the administered doses and formulations of nitrate supplementation across studies, ranging from beetroot juice to mixed vegetable sources, potentially resulting in variable nitrate bioavailability and metabolic conversion rates. Third, the primary mechanism of nitrate action involves vascular and endothelial pathways rather than direct modulation of adipose tissue metabolism. While nitrate-derived NO enhances vasodilation, improves blood flow, and increases mitochondrial efficiency, these effects may not substantially influence total energy expenditure or fat oxidation within the timeframes examined in existing studies. Furthermore, the absence of dietary control and physical activity monitoring in several investigations may have obscured subtle metabolic responses (25). Collectively, these observations suggest that the potential metabolic benefits of dietary nitrate may necessitate longer intervention periods, stricter experimental controls, or synergistic approaches combining nitrate supplementation with caloric restriction or exercise to produce measurable anthropometric effects.

The present analysis has several notable limitations. Several limitations should be acknowledged. First, the total sample size of the included studies was relatively small, which may limited the statistical power of the meta-analysis. Significant heterogeneity was observed among the included studies regarding nitrate dosage, intervention duration, and participant demographics, potentially contributing to outcome variability. The robustness of our findings is constrained by relatively small sample sizes (ranging from 18 to 62 participants per study), brief intervention durations (≤13 weeks), and inconsistent dietary control measures. Moreover, the generalizability of the current evidence is limited due to the predominant inclusion of obese adult male participants, with inadequate representation of female subjects, adolescent populations, and individuals from diverse ethnic backgrounds (26). Nevertheless, this study demonstrates methodological strengths, including its novel contribution as the first meta-analysis to systematically evaluate the effects of dietary nitrate on body weight and associated anthropometric parameters, while some trials exhibited minor concerns regarding allocation concealment and blinding procedures, the overall methodological quality of the studies was high, contributing to the reliability of the pooled estimates. The comprehensive search strategy and inclusion of all available randomized controlled trials through April 1st, 2026 further enhance the validity of this evidence synthesis.

Future investigations should prioritize large-scale, long-term RCTs with durations exceeding 6 months to several years, incorporating stringent control of confounding variables to elucidate the sustained impact of nitrate on vascular and metabolic outcomes (27). Additionally, subgroup analyses stratified by demographic and metabolic characteristics, such as sex, age, and baseline metabolic status, could refine individualized nitrate dosing regimens and intervention durations (28). Further exploration of combined therapeutic strategies, including concurrent administration of nitrate with dietary fiber, antioxidants, or caloric restriction, may uncover potential synergistic effects on cardiometabolic health and weight regulation. Mechanistic studies employing molecular and physiological methodologies are warranted to delineate the role of nitrate-derived nitric oxide in modulating adipose tissue metabolism, skeletal muscle bioenergetics, and tissue perfusion, thereby identifying novel therapeutic targets. Given the current limitations in study quantity and methodological rigor, future high-quality, well-designed, large-scale, and long-term investigations are imperative to clarify the role of nitrate in weight management and to optimize evidence-based nutritional interventions for obesity.

In conclusion, this meta-analysis indicates that dietary nitrate supplementation has minimal impact on anthropometric parameters, including body weight and BMI. However, significant improvements were observed in SBP as well as lipid-related parameters, including TC and TG. These findings suggest that the benefits of dietary nitrate may be more closely related to cardiovascular and metabolic regulation rather than weight reduction. Given the limited number of studies, further high-quality evidence is required to confirm these observations.

## Data Availability

The original contributions presented in the study are included in the article/Supplementary material, further inquiries can be directed to the corresponding authors.
