# Supplementary material for: The effect of dietary nitrate on weight management: a systematic review and meta-analysis
Source: Front Public Health. 2026 Apr 28;14:1798811. doi: 10.3389/fpubh.2026.1798811 (PMC13161109; doi:10.3389/fpubh.2026.1798811)
Supplement: Supplementary file 2 [file Table_1.docx]

Supplementary table 1. Search strategy for included studies.

| MEDLINE via PubMed | #1 "beetroot"[All Fields] OR "beetroot juice"[All Fields]  #2 "nitrates"[MeSH Terms] OR"nitrites"[MeSH Terms] OR"dietary nitrate"[All Fields]  #3 "obesity"[MeSH Terms] OR "overweight"[MeSH Terms] OR "body weight"[MeSH Terms] OR "weight management"[MeSH Terms]  (#1 OR#2) AND #3 AND #4  **Updated Search**  #4 RCT | April 1st  2026 |
| --- | --- | --- |
| Scopus via Elsevier | #1 TITLE-ABS-KEY(beetroot) OR TITLE-ABS-KEY(beetroot juice)  #2 TITLE-ABS-KEY(nitrates) OR TITLE-ABS-KEY(nitrites) OR TITLE-ABS-KEY(dietary nitrate)  #3 TITLE-ABS-KEY(obesity) OR TITLE-ABS-KEY(overweight) OR TITLE-ABS-KEY(body weight) OR TITLE-ABS-KEY(weight management)  #1 AND #2 AND #3 | April 1st  2026 |
| Embase via Elsevier | #1 "beetroot"/exp OR "beetroot juice"/exp  #2 "nitrate"/exp OR "nitrite"/exp OR " dietary nitrate"/ti,ab,kw  #3 "obesity"/exp OR "overweight"/exp OR "body weight"/exp OR "weight management"/exp  (#1 OR #2) AND #3 AND #4  **Updated Search**  #4 'randomized controlled trial'/de | April 1st  2026 |
| Web of Science-Science Citation Index and Social Sciences Citation Index via Clarivate | #1 TS=(beetroot) OR TS=(beetroot juice)  #2 TS=(nitrates) OR TS=(nitrites) OR TS=(dietary nitrate)  #3 TS=(obesity) OR TS=(overweight) OR TS=(body weight) OR TS=(weight management)  #1 OR #2 AND #3 AND #4  **Updated Search**  #4 Article(Document Types) | April 1st  2026 |
