# Supplementary material for: The effect of dietary nitrate on weight management: a systematic review and meta-analysis
Source: Front Public Health. 2026 Apr 28;14:1798811. doi: 10.3389/fpubh.2026.1798811 (PMC13161109; doi:10.3389/fpubh.2026.1798811)
Supplement: Supplementary file 4 [file Table_3.docx]

Supplementary table 3: Detailed risk-of-bias assessment of included randomized controlled trials using the Cochrane RoB 2.0 tool.

| **Study ID** | **Randomization process** | **Deviations from intended interventions** | **Missing outcome data** | **Measurement of the outcome** | **Selection of the reported result** | **Overall risk of bias** |
| --- | --- | --- | --- | --- | --- | --- |
| Alharbi 2023 | Low | Low | Low | Low | Low | Low |
| Asgary 2016 | Low | Low | Low | Low | Some concerns | Low |
| Ashor 2014 | Some concerns | Low | Low | Low | Low | Low |
| Babateen 2023 | Low | Low | Low | Low | High | Some concerns |
| Lara 2015 | Low | Some concerns | Low | Some concerns | Low | Some concerns |
| Smeets 2020 | Low | Low | Low | Low | Low | Low |
| Smeets 2022 | Low | Low | Low | Low | Low | Low |
| Wong 2014 | Low | Low | Low | Low | Low | Low |

Note: Risk of bias was assessed using the Cochrane RoB 2.0 tool across five domains: randomization process, deviations from intended interventions, missing outcome data, measurement of the outcome, and selection of the reported result. Each domain and the overall risk of bias were judged as “low risk,” “some concerns,” or “high risk” according to RoB 2.0 guidance.
